# An Unusual Case of Unilateral Foot Drop and Deep Vein Thrombosis Following Electroconvulsive Therapy in a Middle-Aged Patient With Depression

**DOI:** 10.7759/cureus.99436

**Published:** 2025-12-17

**Authors:** Omotola Ogunjobi, Abimbola R Lawal, Nina Patel, Adebusola Adegbola, Lily H Pilkington

**Affiliations:** 1 General Adult Psychiatry, Black Country Healthcare NHS Foundation Trust, Dudley, GBR; 2 Emergency Medicine, Manchester University NHS Foundation Trust, Manchester, GBR; 3 Psychiatry, Humber Teaching NHS Foundation Trust, Kingston upon Hull, GBR

**Keywords:** dvt and footdrop, electroconvulsive therapy (ect), management of severe depression, physical treatments in psychiatry, post-ect foot drop

## Abstract

Electroconvulsive therapy (ECT) is a well-known, generally safe, and effective treatment modality for severe mental health disorders, including catatonic depression, after other forms of approved treatments have failed. Like most procedures, ECT is known to have some adverse effects, such as transient headaches and transient amnesia; however, neurological complications would be classed as exceptionally rare, especially with the modified ECT.

We report the case of a 54-year-old woman with a recurrent depressive disorder with catatonic features who presented with low mood, reduced energy levels, loss of appetite with refusal of oral intake, intense suicidal ideation and attempts, mutism, psychomotor retardation, and eventual lack of movement.

She was initially stabilized on the medical ward and commenced ECT. After the fourth session, there was an appreciable improvement in her mood and activity levels, and then she complained of weakness in her right foot, which was later confirmed clinically to be a right-sided foot drop.

Despite having had two previous sessions of ECT in past years, this was the first time she was experiencing such a symptom following the procedure. ECT was discontinued, and she is being managed conservatively with leg splints by the trauma and orthopedics team and physiotherapists.

A few days following the sudden foot drop, she complained of swelling in her right ankle and pain in her right calf. Upon examination and investigation, a deep venous thrombosis (DVT) was discovered in the right leg. This is the second time she has had DVT following a catatonic depressive disorder, and she is being managed by the anticoagulation team.

This case highlights an extremely rare but possible complication of ECT, which is foot drop, as there are similar cases highlighted in other literature. Although a direct link between ECT and foot drop and/or DVT has not been established, it is important to explore possible reasons why patients may be at risk of these two conditions.

## Introduction

Electroconvulsive therapy (ECT) is still one of the most effective treatments for severe mental health disorders like severe depressive disorders with catatonia [1]. Generally, it is considered safe, with the most common adverse effects usually limited to transient cognitive disturbances [2]. Peripheral nerve injuries, including foot drop, are extremely rare and certainly underreported in the body of literature available [3]. Apart from the sudden onset foot drop, the subsequent deep venous thrombosis (DVT), although recurrent and likely a consequence of prolonged bed stays and inactivity, makes this case one-of-a-kind. 

## Case presentation

A 54-year-old woman with a known history of recurrent severe depressive disorder with catatonic features was admitted due to an acute deterioration in her mental state after she stopped taking her prescribed medications: olanzapine 15 mg at night and mirtazapine 45 mg at night. She presented at the outpatients' clinic with a steady decline in her mood and energy levels and was encouraged to reinstate medication use. A few days later, contact was made with the crisis team, as she had now become rather negative in her thinking, had stopped eating and drinking, spoke minimally, and had attempted to end her life until she was discovered by a loved one. Upon admission to the hospital, she presented as mute and incontinent for both urine and feces, with extreme psychomotor retardation leading to not walking but occasionally assuming unusual positions (posturing), refusal to eat, and significant weight loss. 

Initial laboratory investigations at the acute hospital revealed normal results except for the acute kidney injury (Table 1) due to dehydration, and this was promptly corrected with intravenous fluids. Due to not eating and refusal to eat, nasogastric (NG) feeding via a tube was initiated to ensure adequate nutrition. She was nursed under one-to-one care. A mental state examination revealed mutism, as she would not respond to any questions and would not gesture or speak at all, catatonic posturing, and stereotypic movements like flapping of hands. 

**Table 1:** Results of the electrolytes, urea, and creatinine for testing renal functioning. eGFR: estimate glomerular filtrate rate

Parameter	Initial values	Reference ranges	Units
Sodium	145	133-146	mmol/L
Potassium	4.3	3.5-5.3	mmol/L
Urea	8.7	2.5-7.8	mmol/L
Creatinine	89	50-98	umol/L
EPI eGFR	63	>59	mL/min/1.73 m^2^

Past psychiatric history included a similar episode of catatonic depression exactly one year earlier, which had resolved after two sessions of ECT. She had also been diagnosed with DVT a few days after the prolonged bed stay, and ECT was done. This was treated by use of the anticoagulant rivaroxaban, which she discontinued a few months prior to the current admission. 

In her current episode, after initial medical stabilization, she underwent bilateral modified ECT under general anesthesia. Each session of ECT produced a slight improvement in her symptoms; however, marked improvement in speech, oral intake, and movement was noted after the third session of ECT. She had begun to eat a few meals, communicate in simple words, and could sit up in bed. The fourth session also produced significant changes as she spoke more, moved around, and was able to feed independently of the NG tube; however, she reported something she felt must have been a complication of the ECT: difficulty in walking. She said that she could not feel sensation apart from ‘pins and needles’ (paresthesia) in her right foot and found that she was dragging along the foot and could not walk as she used to in the past. 

Although most of the team had not heard of foot drop as a complication of ECT, our patient seemed sure it must have caused it, as she never had any problems with her foot prior to the procedure. She then requested to stop the ECT sessions. Due to significant improvements in her mental state and having the capacity to make decisions regarding her treatment, ECT was stopped. 

In total, she had four sessions of ECT done in two weeks with a 72-hour interval between each session. A referral was made to the trauma and orthopedics team and the physiotherapy team for co-management. 

**Figure 1:**
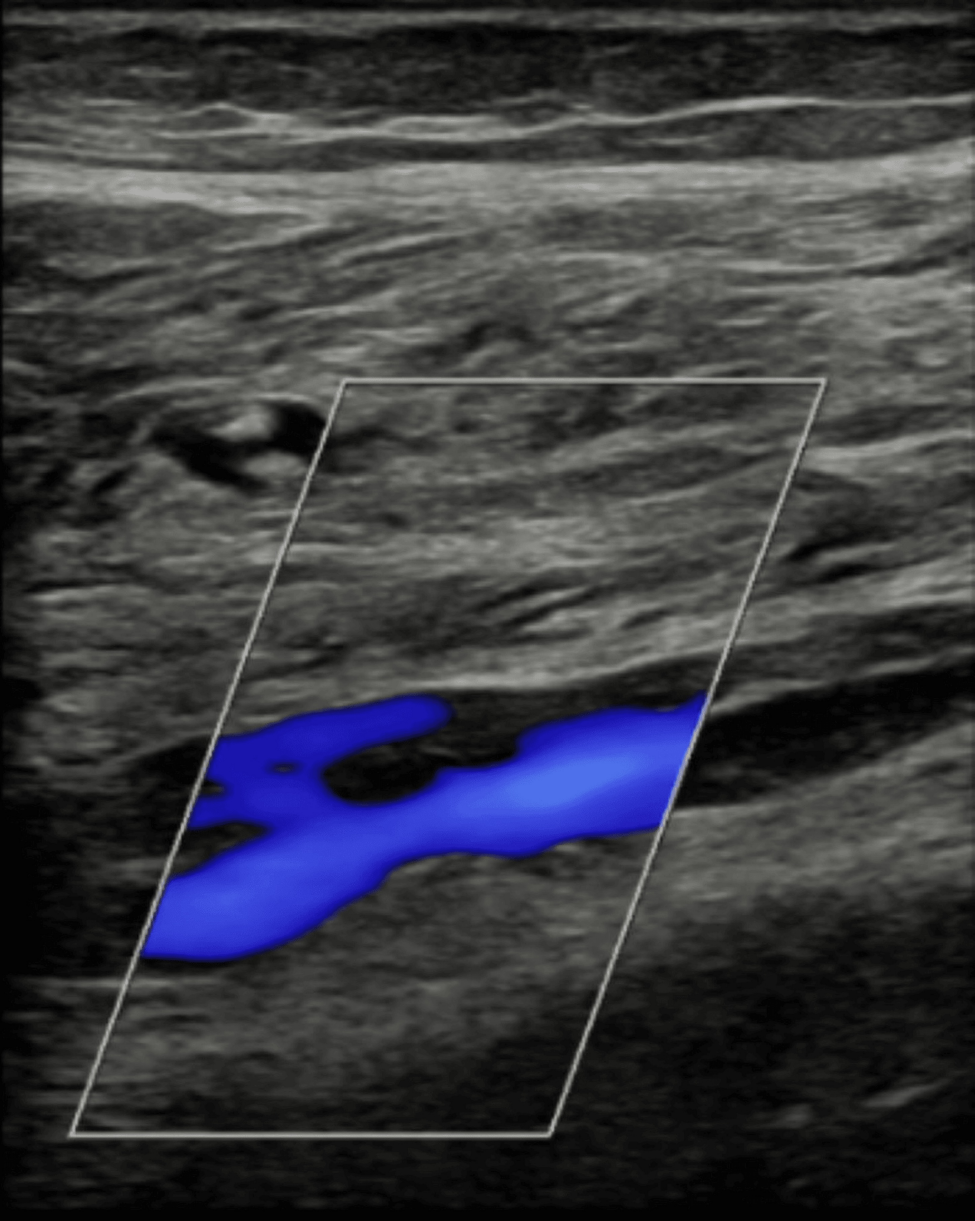
Shows a USS image of the right distal peroneal vein where a right-sided non-occlusive DVT was visualized. USS: ultrasonography scan; DVT: deep venous thrombosis

**Figure 2:**
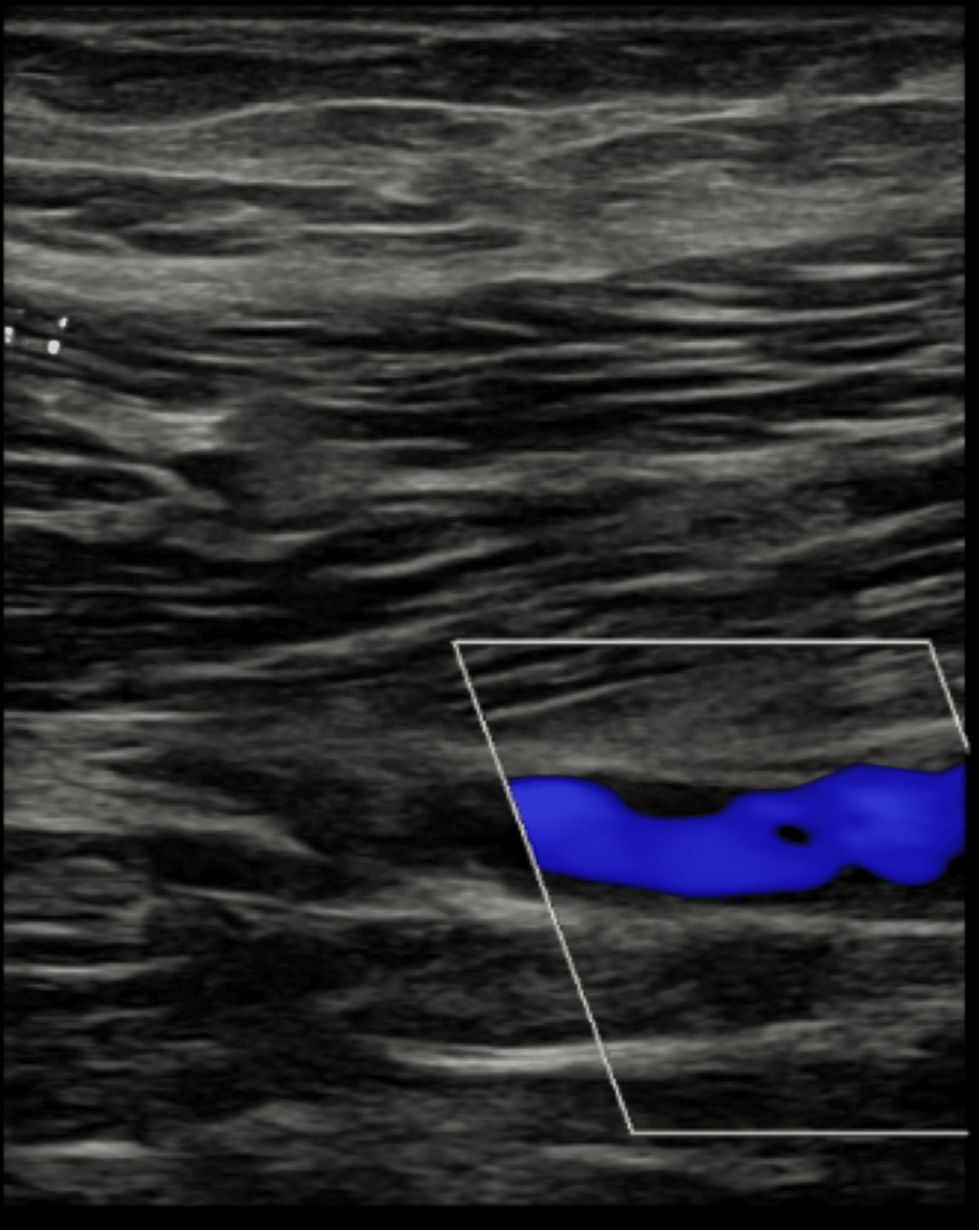
Shows a USS image of the left distal superficial femoral vein where a chronic DVT was visualized. USS: ultrasonography scan; DVT: deep venous thrombosis

**Figure 3:**
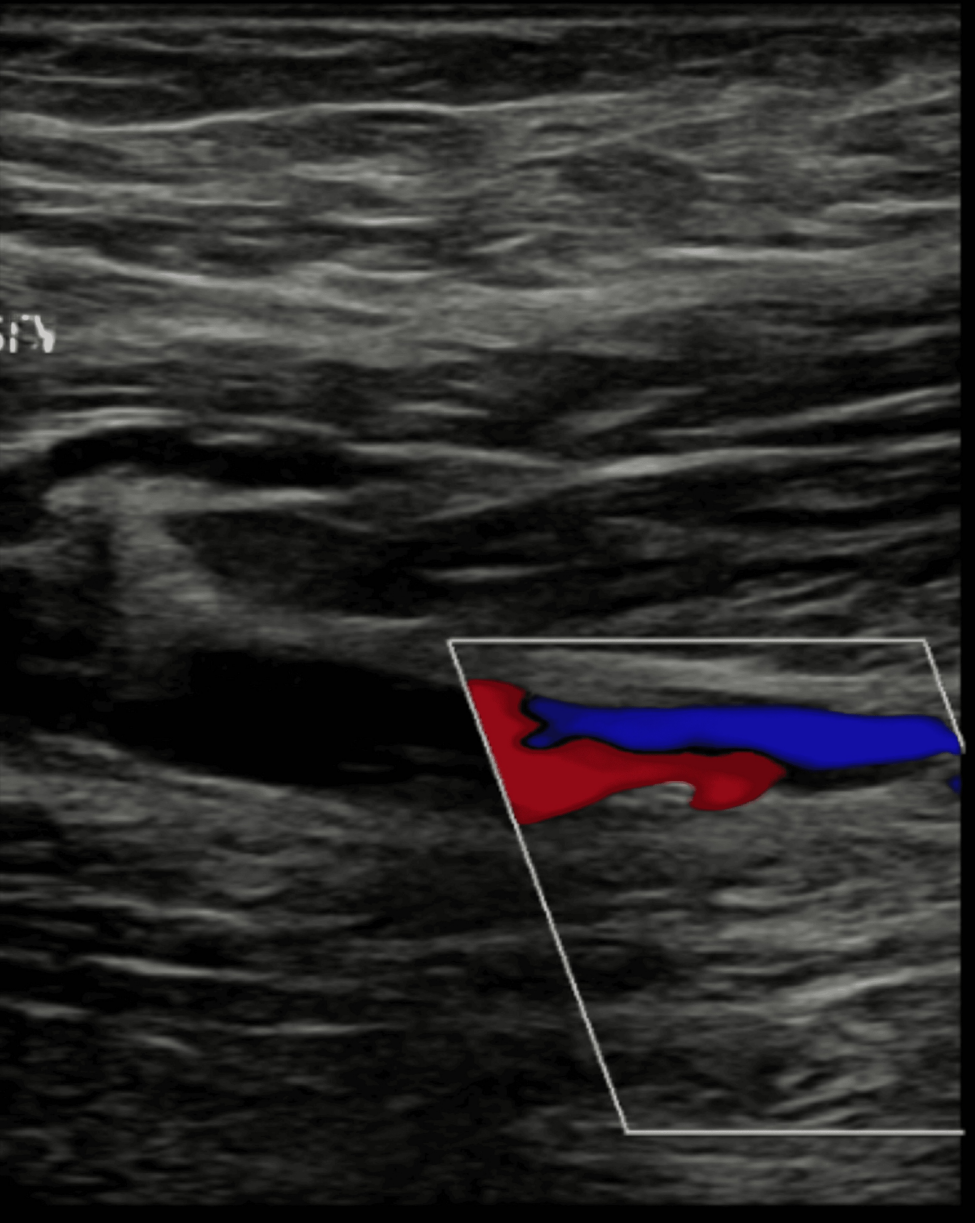
Shows another USS image of the left distal superficial femoral vein where a chronic DVT was visualized. USS: ultrasonography scan; DVT: deep venous thrombosis

A few days after the foot drop complaint, she also complained of pain in the right calf. Upon examination, the right calf was tender, and pedal edema up to the mid-shin was noted. She then volunteered a previous history of DVT in her last mental health admission, which was managed with the use of anticoagulants. This raised high suspicions of another DVT, and urgent investigations, including an ultrasonography of the lower limbs, revealed a DVT in the right leg (Figures 1-3).

There was no history of trauma, diabetes, alcohol use, or prior neurological symptoms. This was the first episode of foot drop in her life, and no mechanical cause could be identified, save for possible prolonged compression of the peroneal nerve during the catatonic episode. 

**Figure 4:**
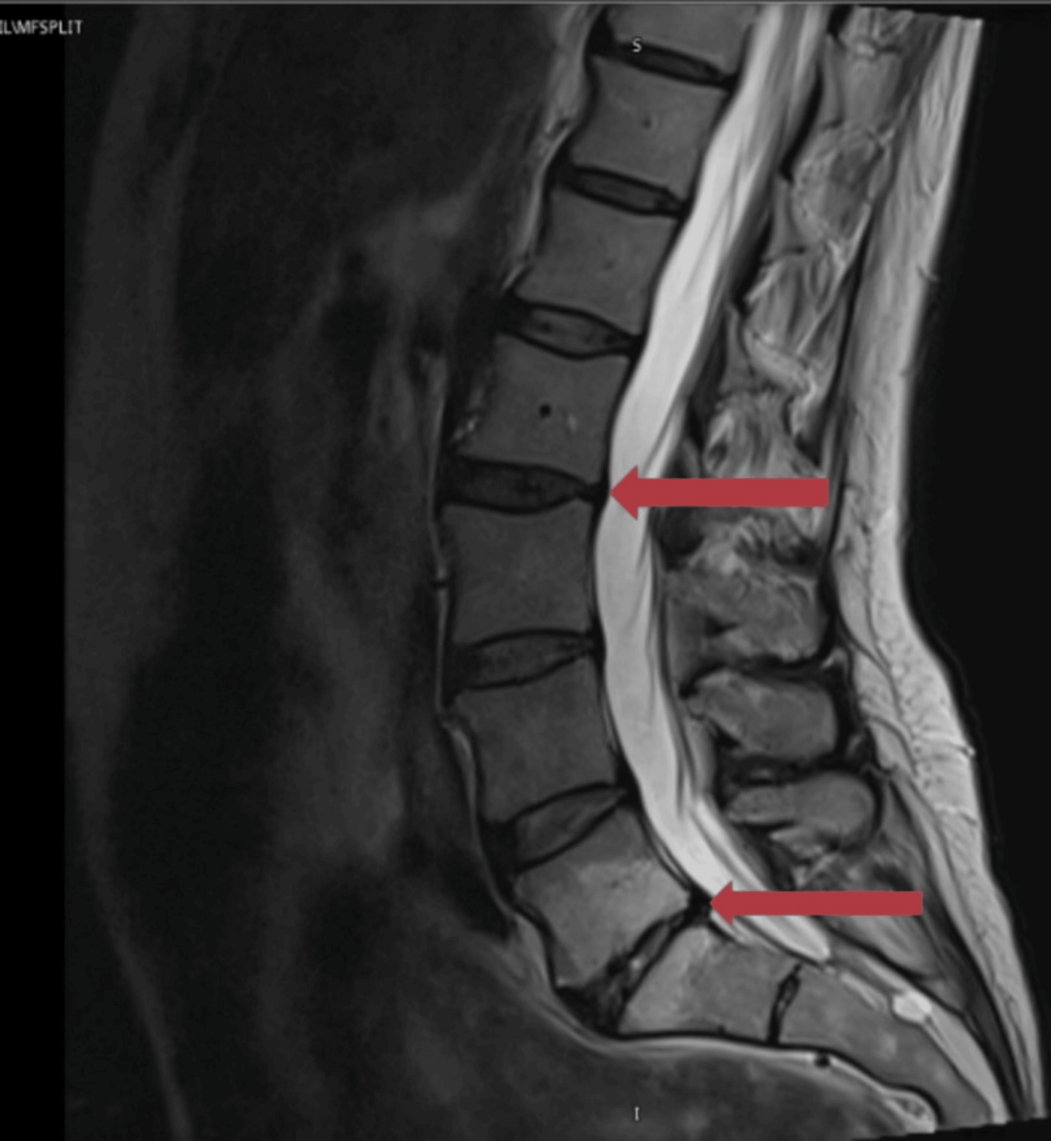
MRI image of the spine (lumbar and sacral region). The red arrows indicate areas where there are minor degenerative changes with some loss of disc height, but there is no substantial vertebral misalignment. There was no frank neural impingement and no other pertinent positive findings identified. The report concluded that there was no discrete structural cause for the reported clinical presentation identified.

The probable causes of the sudden foot drop include common peroneal nerve compression (likely due to postural factors such as prolonged immobility); central neurological lesion, which was ruled out by imaging (Figure 4); functional neurological disorder; drug-induced or metabolic neuropathy (none identified); and nutritional deficiency neuropathy (laboratory tests do not point to this) (Tables 2-9).

**Table 2:** Full blood count revealing mild lymphocytopenia and no other obvious abnormalities. Hb: hemoglobin; WBC: white blood cell; PLTS: platelets; RBC: red blood cell; Hct: hematocrit; MCV: mean corpuscular volume; MCH: mean corpuscular hemoglobin; MCHC: mean corpuscular hemoglobin concentration; NRBC: nucleated red blood cell

Parameter	Initial values	Reference ranges	Units
Hb	138	115-165	g/L
WBC	7.40	4-11	10*9/L
PLTS	376	150-450	10*9/L
RBC	4.74	3.8-5.8	10*12/L
Hct	0.427	0.37-0.47	L/L
MCV	90.1	80-100	fL
MCH	29.1	27-32	pg
MCHC	323	320-360	g/L
NRBC	0.00	0-0.2	10*9/L
Neutrophils	5.26	2-7.5	10*9/L
Lymphocytes	1.42	1.5-4.5	10*9/L
Monocytes	0.71	0.2-0.8	10*9/L
Eosinophils	0	0-0.4	10*9/L
Basophils	0.01	0-0.1	10*9/L

**Table 3:** Liver function test. Values are within the normal range. ALP: alkaline phosphatase; ALT: alanine aminotransferase

Parameter	Initial values	Reference ranges	Units
Albumin	43	30-45	g/L
Total bilirubin level	7	5-26	umol/L
ALP	97	30-130	IU/L
ALT	36	0-55	IU/L

**Table 4:** Bone profile. No obvious abnormalities.

Parameter	Initial values	Reference ranges	Units
Total calcium	2.38	2.2-2.6	mmol/L
Adjusted calcium	2.37	2.2-2.6	mmol/L

**Table 5:** Vitamin B12 and folate levels. Values are within the normal range.

Parameter	Initial values	Reference ranges	Units
Vitamin B12	68	51-128	pmol/L
Folate	6.1	3.1-20.5	ug/L

**Table 6:** Thyroid-stimulating hormone (TSH) values. Values are within the normal range.

Parameter	Initial values	Reference ranges	Units
TSH	1.78	0.35-4.94	mIU/L

**Table 7:** Prolactin levels. Values are within the normal range.

Parameter	Initial values	Reference ranges	Units
Total prolactin	331	63-561	mIU/L

**Table 8:** Lipid profile. Increased total cholesterol levels and LDL cholesterol. LDL: low-density lipoprotein; HDL: high-density lipoprotein

Parameter	Initial values	Units	Reference ranges
Total cholesterol	5.9	mmol/L	= 5mmol/L
HDL cholesterol	1.9	mmol/L	>/= 1.2mmol/L (women)
Non-HDL cholesterol	4.0	mmol/L	= 4mmol/L
Cholesterol ratio	3:1		= 4mmol/L
Triglycerides	0.9	mmol/L	<1.7mmol/L
LDL cholesterol	3.6	mmol/L	= 3mmol/L

**Table 9:** Serum levels. Serum levels of magnesium (normal levels), slight increase in phosphate levels, and a significant increase in creatinine kinase levels.

Parameter	Initial values	Reference ranges	Units
Magnesium	1.00	0.7-1.0	g/L
Phosphate	1.55	0.8-1.5	umol/L
Creatine kinase	3630	29-168	IU/L

Other investigations, such as MRI of the head (image not available), revealed no focal signal or structural abnormality, no mass lesion, collection, or vascular territory infarction. There was no demyelination, no restricted diffusion. The lateral ventricles are normal, and the sulci and basal cisterns are clear. The cerebellum and brainstem were normal, as were the normal orbits and midline structures. A conclusion of an unremarkable examination was made.

Overall, she was diagnosed with recurrent depressive disorder (current episode severe depressive disorder with psychotic symptoms), F33.3 (The International Classification of Diseases-10 (ICD-10) of Mental and Behavioral Disorders) [4], and catatonic features, along with unilateral common peroneal nerve compression of the right foot and DVT in the right leg.

Treatment and outcome 

After four sessions of ECT, a considerable improvement in this patient's catatonic symptoms was noticed. However, at this point, the patient raised concerns that the ECT had caused the foot drop impairment and declined further sessions. The physiotherapy team reviewed this patient, recommended exercises, and fitted her with a leg splint to provide foot drop support. She was then stabilized on mirtazapine 45 mg at night and olanzapine 15 mg at night and continued to demonstrate notable improvement. She remains on long-term rivaroxaban 20 mg once-daily treatment, as per the anticoagulation clinic protocol. This patient’s foot drop and pain remained consistent from the onset of this issue, as co-codamol provided minimal relief from the pain caused. The general practitioner and trauma and orthopedics teams were allowed to take up the further management of the foot drop.

## Discussion

Methodology 

A range of databases and websites were searched, including Ovid Medical Literature Analysis and Retrieval System Online (MEDLINE), Ovid Excerpta Medica Database (Embase), Ovid APA Psychological Information Database (PsycInfo), Cumulative Index to Nursing and Allied Health Literature (CINAHL Ultimate), PubMed, Ovid Emcare, and Google Scholar.

Evidence searches for “Foot Drop Following Electroconvulsive Therapy Procedure [post- ECT]” yielded five articles; three of the five are case reports. The oldest goes back to 1958. For the evidence search for “foot drop as a presentation of deep vein thrombosis,” two case reports were found. Other search terms included "foot drop and DVT" and "occurrence of DVT after ECT."

Analysis

ECT has been in clinical use for more than 80 years [5]. Despite its long history, it remains a subject of considerable controversy, contributing to its underutilization as a treatment modality [6]. Advances in psychiatry have transformed ECT from the crude “electric shock” procedures of the 1930s into a more refined intervention associated with fewer side effects and a more patient-centered approach [7]. It is one of the safest procedures and is now performed under general anesthesia [6]. 

ECT evolved from the concept of chemically induced seizures. Based on clinical observations, Laszló Meduna proposed an antagonistic relationship between epilepsy and schizophrenia, and after several experiments, he successfully induced seizures using camphor [5]. The first convulsive therapy was administered on January 2, 1934, and by 1938, electrical stimulation had replaced chemical induction, with the first ECT performed on April 11 of that year [5]. 

ECT was initially administered without general anesthesia, a practice referred to as “unmodified ECT.” This approach carried significant risks, including vertebral, dental, and limb fractures; cardiovascular complications such as bradycardia and asystole; and psychological trauma [8]. In 1950, “modified ECT” was introduced in the United Kingdom, UK. Modified ECT is ECT incorporating general anesthesia and muscle relaxants, and by the early 1960s, all ECT administered in the UK had adopted this approach [8]. 

Well-documented side effects of modified ECT range from common to uncommon. Common side effects include transient memory loss, headaches, nausea, and muscle soreness. While uncommon, serious side effects might include persistent memory impairment, cardiovascular effects, and fractures. There are also other immediate potential complications, such as status epilepticus, laryngospasm, and peripheral nerve palsy, which overall have an estimated incidence of one per 1,300 to 1,400 treatments [8]. Most of these side effects are temporary, and a thorough pretreatment assessment, consent, and monitoring during and after ECT, as outlined in National Institute for Health and Care Excellence (NICE) guidelines [8], are essential to mitigating potential side effects. 

ECT involves the application of an electric current to induce a generalized cerebral seizure [9]. It is primarily indicated when rapid and short-term relief is required, particularly in cases where other treatments have failed or the condition poses a significant risk to life. Indications include severe depressive illness, catatonia, prolonged or severe manic episodes, and, in selected cases, schizophrenia [9]. 

Foot drop, also referred to as common peroneal palsy or neuropathy, is characterized by an inability to dorsiflex the foot due to weakness of the dorsiflexor muscles [10]. Peroneal neuropathy represents the most frequent compressive neuropathy of the lower limb [11]. The common peroneal (fibular) nerve arises from the posterior divisions of the L4-S2 nerve roots as part of the sciatic nerve; this bifurcates into the tibial and peroneal components proximal to the popliteal fossa. The common peroneal nerve courses posterolaterally, lying just behind the long head of the biceps femoris, and then winds around the fibular neck, a superficial location that renders it particularly vulnerable to injury, and then continues into the lateral compartment of the leg. At or near the fibular neck, it divides into deep and superficial branches [12,13]. The deep peroneal nerve travels between the tibialis anterior and extensor hallucis longus muscles within the anterior compartment, continuing along the anterior tibia before terminating in the first dorsal webspace. The superficial peroneal nerve descends through the lateral compartment of the leg and terminates on the dorsum of the ankle and foot [13]. 

Compressive etiologies represent the most common cause for peroneal nerve palsy [10]. Non-mechanical causes include inherited or systemic conditions such as Charcot-Marie-Tooth disease and polyarteritis nodosa, although diabetes mellitus remains the most frequent systemic contributor [12]. 

Risk factors for peroneal neuropathy are predominantly traumatic, followed by behavioral. Traumatic causes, such as knee dislocation or fibular fracture, directly damage the nerve and are associated with poorer outcomes. Behavioral causes include prolonged leg crossing or repetitive squatting, which can lead to an acute presentation of peroneal nerve palsy. Significant weight loss is also a recognized risk factor; in one case series, 20% of 150 individuals who lost an average weight of 10.9 kg experienced peroneal nerve neuropathy. Bilateral cases have been observed following extreme weight loss, including 10% of World War II prisoners who lost 5-11 kg [11]. Prolonged immobility, such as in comatose or ventilated patients, and intraoperative positioning without adequate padding of bony prominences, particularly the fibular head, may also result in compressive neuropathy [13]. 

Clinically, foot drop may present partially or completely and may develop over days to weeks [10,13]. The hallmark finding in common peroneal nerve injury is weakness of ankle dorsiflexion, producing foot drop and a high-stepping gait. Sensory disturbances, including numbness or paresthesia over the lateral leg, dorsum of the foot, and first webspace, may accompany motor weakness. Pain may be present in traumatic cases, but it is not universally observed [13]. 

In a study by Elpern et al., three cases of foot drop associated with ECT were observed. In all instances, ECT was applied without glissando (a technique that involves a slower onset of seizure rather than a sudden push technique) or relaxant drugs, and with restraint by two assistants who held the upper extremities in adduction and the patient’s hands on the hips; there was no restraint on the lower extremities [14]. 

Elpern et al. concluded that during an electrically induced convulsion, the feet usually invert and plantarflex strongly and that the muscles in the leg are a part of the generalized muscular contractions and that it is possible that this can produce a compression and traction effect upon the common peroneal nerve, resulting in transient interruption of function, which, in these cases, is mainly motor [14]. 

ECT needs to be safe and effective, with the highest standards of care maintained consistently and independently monitored. The key area of international variation is whether ECT is administered as a modified or unmodified treatment. In the UK, ECT cannot be carried out without general anesthesia. Internationally, the situation is varied. In Denmark, Norway, and Sweden, ECT is modified, but in Thailand, 94% of ECT is delivered unmodified. A survey throughout Russia in 2005 found that fewer than a fifth of ECT was modified. Across much of Asia, unmodified ECT was used in over 90% of the countries, as well as in some countries in Africa and Latin America [8]. 

O’Shea et al., in the case of bilateral foot drop following bilateral ECT, could not find any explanation for the foot drop. Causes such as unusual force being exerted on patients’ knees (neurapraxia) during ECT and evidence of ischemia of the lower limbs or of a metabolic disorder were reasonably ruled out. As it was not a unilateral foot drop, a case could not be made for leg-crossing as a cause. The conclusion by O’Shea et al. was that the case may well be related to ECT itself (being bilateral instead of unilateral), pressure exerted during the procedure, bed rest, poor nourishment, and/or sedation. No definite cause or explanation was identified [15]. 

A report by O’Shea revealed compression as a reason for right-sided foot drop in a patient who developed right foot drop between ECT treatments. It was later discovered that the patient had fallen asleep with his right popliteal fossa dangling over the back of a bench prior to the development of the foot drop [16]. 

Another case report in literature by Raj et al. records bilateral foot drop and double incontinence prior to the start of bilateral ECT treatment for major depression. In this case, the patient was noted to have been sitting cross-legged for hours and developed left foot palsy with high-stepping gait and numbness over the left lateral aspect of his calf and a milder right foot palsy. It was concluded after several investigations and discussions with the neurologist that this was pressure palsy, and the ECT treatments were started. By the end of his ECT treatments, his gait, alongside his other symptoms, was improving. This instance highlights a reversal of symptoms, including foot drop, after ECT [17].

Foot drop can be a rare presentation of DVT. In a case report by Nawaf et al., the patient presented with pain in the left leg, weakness, decreased sensation in the left foot, and a left foot drop. Ultrasound confirmed thrombi in the left external iliac, common, and superficial femoral veins. The left popliteal, posterior tibial, and proximal greater saphenous veins were patent. Investigations, except for D-dimer and ultrasound, were unremarkable, and symptoms of foot drop resolved by the tenth day of treatment [18].

A case of sciatic neuropathy (involving the tibial branch more severely than the peroneal branch) caused by compression due to vascular engorgement associated with DVT was reported by Young et al. The patient was being treated for DVT before the onset of asymmetrical limb weakness and paresthesia. Investigations revealed symptoms to be due to venous reflux resulting from engorgement of the veins around the tibial branch of the sciatic nerve [19]. 

In general, the peroneal branch of the sciatic nerve is more susceptible to and more severely affected by traumatic and compressive injuries than the tibial branch. The tibial branch is, however, more vulnerable to venous reflux compared with the peroneal branch because the latter is supplied by smaller vessels and is not as severely affected by reflux [19]. 

## Conclusions

This is a unique case of unilateral foot drop/common peroneal nerve compression following the fourth session of ECT. What makes this case even more peculiar is the accompanying DVT following complaints of foot drop. In this case, it is difficult to conclude that the footdrop was a direct result of the ECT done. Although there are a few reports of post-ECT foot drop, there is yet to be an established link between having had an ECT and developing a common peroneal nerve palsy. More studies are required to further investigate whether there is a relationship or not.
